# The Selah Pilot Study of Spiritual, Mindfulness, and Stress Inoculation Practices on Stress-Related Outcomes Among United Methodist Clergy in the United States

**DOI:** 10.1007/s10943-023-01848-x

**Published:** 2023-06-26

**Authors:** Rae Jean Proeschold-Bell, David E. Eagle, Logan C. Tice, Jia Yao, Joshua A. Rash, Jessica Y. Choi, Beth Stringfield, Sofia M. Labrecque

**Affiliations:** 1 Duke Global Health Institute, Duke University, Durham, USA; 2 Duke Center for Health Policies and Inequalities Research, Duke University, Durham, USA; 3 Department of Psychology, Memorial University of Newfoundland, St. John’s, NF Canada

**Keywords:** Mindfulness, Stress, Religion, Psychological distress, Prayer, Outcomes, Psychosocial intervention, Health behavior, Anxiety

## Abstract

The job–demand–control–support model indicates that clergy are at high risk for chronic stress and adverse health outcomes. A multi-group pre-test–post-test design was used to evaluate the feasibility, acceptability, and range of outcome effect sizes for four potentially stress-reducing interventions: stress inoculation training, mindfulness-based stress reduction (MBSR), the Daily Examen, and Centering Prayer. All United Methodist clergy in North Carolina were eligible and recruited via email to attend their preferred intervention. Surveys at 0, 3, and 12 weeks assessed symptoms of stress, anxiety, and perceived stress reactivity. Heart rate variability (HRV) was assessed at baseline and 12 weeks using 24 h ambulatory heart rate monitoring data. A subset of participants completed in-depth interviews and reported skill practice using daily text messages. Standardized mean differences with 95% and 75% confidence intervals were calculated for the change observed in each intervention from baseline to 3 and 12 weeks post-baseline to determine the range of effect sizes likely to be observed in a definitive trial. 71 clergy participated in an intervention. The daily percentage of participants engaging in stress management practices ranged from 47% (MBSR) to 69% (Examen). Results suggest that participation in Daily Examen, stress inoculation, or MBSR interventions could plausibly result in improvement in stress and anxiety at 12 weeks with small-to-large effect sizes. Small effect sizes on change in HRV were plausible for MBSR and Centering Prayer from baseline to 12 weeks. All four interventions were feasible and acceptable, although Centering Prayer had lower enrollment and mixed results.

## Introduction

Experiencing stress at levels detrimental to health is common, yet how to manage stress and its concordant symptoms is still illusive for many people. We sought to assess the feasibility, acceptability, and range of potential effects of four sets of potentially helpful practices, including two spiritual practices. We chose a mix of spiritual and non-spiritual practices for our study population of an occupational group of clergy, who have been shown to have above-average rates of diseases including hypertension, diabetes, arthritis, angina, and asthma (Proeschold-Bell & LeGrand, 2010) and to encounter numerous work-related stressors (Proeschold-Bell et al., 2011).

Physiological stress responses occur when one experiences a stressor and perceives that the demands exceed one’s personal and social resources (Lazarus & Folkman, 1984). Brief bouts of stress can be protective, but chronic stress can take a toll through allostatic overload (McEwen & Gianaros, 2011). Chronic stress has been associated with a vast array of diseases from metabolic syndrome, including weight gain, dyslipidemia, type 2 diabetes, and hypertension (Bergmann et al., 2014), to a moderately elevated (i.e., 10–40%) risk of heart attack and stroke (Kivimaki & Kawachi, 2015). It is particularly important to reduce allostatic overload among people with chronic diseases to prevent further deterioration.

Chronic stress is common. Thirty-five percent of United States (US) adults indicated being extremely stressed over the last month, and almost one-third reported attending a doctor’s appointment for stress-related complaints (Everyday Health, 2019). Clergy report high levels of stress; a literature review of clergy mental health articles from 1975 to 2000 found high levels of occupational stress across denominations and attributed the stress to “extraordinary demands,” criticism, congregational conflicts, and expectations of clergy family members (Weaver et al., 2002, p. 398). A more recent scoping review of Catholic priests attributed stress to work overload, lack of boundaries, and perfectionistic personality styles (Ruiz-Prada et al., 2021). Clergy exhibit physical health indicators of chronic stress, including diabetes, hypertension, asthma, joint-related disease, cardiovascular disease, and obesity (Baruth et al., 2014; Halaas, 2002; Proeschold-Bell & LeGrand, 2010). Further, studies indicate high rates of anxiety among clergy (e.g., Knox et al., 2002; Lau, 2018; Proeschold-Bell et al., 2013) and above-average rates of depression compared to non-clergy (Knox et al., 2002; Proeschold-Bell et al., 2013).

The job–demand–control–support (JDCS) model indicates that stressful jobs are characterized by high demand, low control, and low support (Van der Doef, 1999). Requiring a broad skill set (DeShon, 2012), clergy perform many demanding roles, including inspiring the congregation, providing one-on-one care for congregants, performing sacraments, educating congregants, overseeing educational programming, leading social justice activities, and attending to unexpected needs and conflict (Kuhne & Donaldson, 1995). The work week typically averages 50 h or more with the expectation of being on call around-the-clock (Carroll, 2006). Clergy direct a mainly volunteer workforce and, with the wide range of tasks conducted, often do not receive the support needed to match the tasks or the emotional challenges faced (Morris & Blanton, 1998; Proeschold-Bell, 2018).

The clergy profession is a prime example of having a calling with unbounded work hours. The ambiguity of which direction to take and which needs to prioritize are stressors for clergy. Even though clergy report a strikingly high degree of satisfaction with work (Smith, 2007; Stewart-Sicking, 2009), which can be deeply meaningful and thus life-enriching (Johnson & Jiang, 2017), they also frequently report emotional exhaustion and a lack of personal accomplishment (Adams & Bloom, 2017). Sixty-one percent of Catholic Polish priests believe their ministry has had a detrimental effect on their health (Kalita et al., 2023). Many clergy prioritize caring for others ahead of their own well-being, which may increase their risk of harm from stress (Rogers, 2022). In this way, clergy are similar to other employees who are called to their work (e.g., medical providers, first responders, and social workers) and could benefit from stress management practices.

Researchers have developed numerous approaches to manage stress. The most prominent approaches include aspects of cognitive-behavioral therapy, mindfulness, and relaxation (Varvogli, 2011). Stress-reducing activities are viewed as skills that require regular practice (Rao et al., 2013; Walton, 2002). As such, the most effective interventions are those that individuals are willing and motivated to practice (i.e., patient preferences are an important aspect of evidence-based practice (Spring, 2007)). A recent study found that only half of clergy with elevated anxiety or depressive symptoms sought professional help (Biru et. al, 2023). Pilot and feasibility study data can provide important information about occupational workers and their willingness to engage in stress management practices. For example, 83% of newly registered nurses in a pilot study were willing to engage in 4 h of training on proactive, protective behaviors such as asking for help (Frögéli et al., 2018), and early elementary school teachers attended 87% of 27 h of training in mindfulness-based stress reduction, but suggested shortening it and making more explicit links to their teaching (Braun et al., 2020).

Following best practices for developing behavioral treatments (Czajkowski et al., 2015), we conducted Phase-II preliminary testing of four potentially stress-reducing interventions to: 1) evaluate the feasibility and acceptability of the trial protocols; 2) inform intervention modifications; and 3) provide initial estimates of effect needed to design an adequately powered Phase III efficacy trial. The goal of this pilot intervention study was to determine interest in each of four interventions that we believed would be acceptable to clergy, collect data to inform modification of intervention content and delivery, and identify trends in outcomes to inform an adequately powered trial evaluating the most promising stress-reduction interventions.

The interventions included two spiritual practices: the Daily Examen and Centering Prayer. We also tested an intervention combining diaphragmatic breathing techniques with stress inoculation training. Finally, we included mindfulness-based stress reduction (MBSR) as a gold standard stress-management intervention shown to provide changes in both self-reported (Shapiro et al., 2005) and biometric indicators of stress (Krick et al., 2021).

## Methods

### Study Population

Eligibility was based solely on occupational status. All appointed clergy members in July 2018 of the North Carolina Annual Conference and the Western NC Annual Conference of the United Methodist Church (UMC) were eligible to participate and identified through conference documents. No stress or health status inclusion criteria were used. A total of 1,642 clergy were eligible to participate, and we sought 70–120 for this pilot study.

### Study Design and Recruitment

We employed a multi-group pre-test–post-test nonrandomized participant preference design. Recruitment involved an extensive communication campaign using a mailed paper invitation, email, UMC electronic newsletters, and in-person appeals at clergy meetings. Interested clergy were directed to a website where, after consenting online, clergy provided demographic information and chose an intervention workshop to attend (two date/location options were provided for each workshop; interventions occurred at small retreat centers). Clergy intervention preference included a combination of the intervention content, dates, and location.

The Duke University Campus Review Board approved all procedures (protocol #2019–0238) and participants gave free and informed consent.

### Interventions

#### Mindfulness Based Stress Reduction (MBSR)

We included an option to participate in MBSR as a gold standard for stress reduction (Grossman, 2010; Kabat-Zinn, 2013). MBSR uses secular meditation techniques to train attention to one’s thoughts and body to encourage cognitive appraisal to stressors and reduce emotional reactivity (Teasdale, 1995). The MBSR curriculum of 8 weekly sessions was conducted via videoconference with a phone option by trained MBSR instructors, using the national model first developed by Jon Kabat-Zinn (2013). It included exercises in awareness of breath, body scans, walking meditation, “choiceless” open awareness, Loving Kindness Meditation, and bringing awareness to the present moment. An optional, in-person Day of Mindfulness was included. Participants were encouraged to practice for 45–60 min/day the content covered in that week’s class for the first 8 weeks, and for an additional 4 weeks, formal practice of any of the exercises.

#### Stress Proofing

We included an intervention called Stress Proofing, which is a set of multiple stress reduction skills with aspects of stress inoculation training (Meichenbaum, 2007), curated, combined, and delivered by the founder of an organization called NCSystema. Two people from NCSystema designed and led a two-day, overnight workshop with 11 h of content. Consistent with stress inoculation training, the workshop content began with education on how people respond to stress, followed by ways to become aware of when and how one responds to stress and how the effects of stress can linger in the body (Lazarus & Folkman, 1984; Meichenbaum, 2007).

The training then diverged from traditional stress inoculation training and focused on physical activities to undo the stress response. These activities included walking with diaphragmatic breathing, triangle and rectangle breathing, tension control, stretching, and massaging the muscles around the ribcage near the vagus nerve where tension is often held. The workshop included a discussion on stress inoculation training, encouraging participants during periods of less stress to allow themselves a degree of physical discomfort to learn to tolerate discomfort in preparation for future stressors (Meichenbaum & Cameron, 1983). As an example, participants laid down on itchy, wet grass for five minutes. The workshop content also included a variety of lifestyle recommendations such as prioritizing nutrition and sleep and disengaging from technology for several hours before sleep. The daily practice plan emphasized stress awareness and diaphragmatic breathing, with encouragement to try the lifestyle adjustments for a few days at a time.

We recommended that participants practice the skills learned for 10–25 min per day for three months. We gave participants a book created for the study that covered the information learned in the workshop and provided a daily practice plan for each of 30 days, beginning immediately after the workshop. Three weeks after the workshop, one instructor offered a one-hour class session via videoconference.

#### Daily Examen (Examen)

The Examen is a Jesuit prayer practice (Thibodeaux, 2015) that we chose because an earlier qualitative study of ours found that clergy with high levels of positive mental health and low levels of burnout handle criticisms through asking if and how they relate to their current mission, a practice which they conceptualized as working in alignment with God (Case et al., 2020). If the criticism did not relate to their current mission, clergy could more easily move on from it. Also, experience from the Spirited Life study indicates that clergy have difficulty being self-compassionate toward themselves (Proeschold-Bell et al., 2017), which may increase stress symptoms (Homan & Sirois, 2017). The Examen seemed a promising brief intervention to help clergy work in alignment with God through its five steps: 1) Become aware of God’s presence; 2) Give thanks to God for everything in your life; 3) Review the events of the day guided by the Holy Spirit; 4) Look at what went well or wrong in the past day; if at fault, ask God for forgiveness; and 5) Look toward to tomorrow—what one thing should you do? Listen to what God is telling you.

Two experts on the Examen designed and led a one-day workshop with 5 h of content, which included 2, 15-min Examen practice sessions. The lead instructor also offered 2, 1-h follow-up sessions with cohorts of up to 4 participants on a videoconference approximately 2 and 4 weeks after the workshop. We recommended that participants practice the Examen, which typically takes 10–15 min, every day for 3 months.

#### Centering Prayer

We chose Centering Prayer because it is a meditative practice, which generally have been shown effective at reducing stress (Liza, 2011), and we thought the spiritual aspects of Centering Prayer might make it acceptable to clergy. Few studies on Centering Prayer exist, but one reported promising reductions in perceived stress and anxiety symptoms (Hayter et al., 2019), and another found that participants became more aware of God’s presence in their lives (Johnston, 2016). An expert in Centering Prayer designed and led a 1-day workshop with 3.5 h of content, which included 2, 20-min Centering Prayer practice sessions. Participants were taught to set a signal, such as a chime alarm before beginning practice and then to sit quietly and empty their mind of thoughts, framed as time to “rest in the arms of God.” If intrusive thoughts arose, they were instructed to return to their chosen word (e.g., “peace”). We also offered a single, 1-h follow-up class videoconference session with the instructor 3 weeks after the workshop. We recommended that participants practice Centering Prayer for 20 min per day for 3 months.

### Measures and Apparatus

Our measures were designed to learn about feasibility, acceptability, preference, and suggestions for improving the interventions. In addition, we sought to evaluate a range of potential intervention effects on the stress response. Per best practice recommendations for psychosocial intervention trials (Crosswell & Lockwood, 2020), we included both self-report and biomarker measures, specifically measuring stress and anxiety symptoms and heart rate variability (HRV). Participants were asked to complete online surveys at baseline (Time 1; usually immediately pre-workshop but possibly up to 6 days later), 3 weeks (Time 2), and 12 weeks (Time 3). They were also asked to provide heart rate variability (HRV) data at Time 1 (immediately post-workshop) and Time 3. Participants were not compensated for their baseline survey and received $25 for each additional survey, as well as $25 per HRV assessment.

Each intervention was received by two cohorts. As a measure of intervention uptake, all participants were sent a daily text message for 12 weeks asking for the number of minutes practiced the prior day. Improvements in the text messaging system enabled the use of data from participants in the second cohort only.

#### Survey Measures

The Calgary-Symptoms of Stress Inventory (C-SOSI) is a 56-item, 8-subscale measure of the frequency of self-reported stress symptoms over the past week (Carlson & Thomas, 2007). We included 5 of the 8 subscales, specifically the subscales of anger (8 items; e.g., “easily annoyed and irritated”), muscle tension (8 items; e.g., “excessive tension, stiffness, soreness or cramping in the muscles in your shoulders”), cardiopulmonary arousal (6 items; e.g., “rapid breathing”; “irregular heartbeats” while not exercising), neurological/gastroenterological (10 items; e.g., “nausea”), and cognitive disorganization (9 items; e.g., “how often does it seem your thinking gets mixed-up when you have to do things quickly?”). We did not include the subscale of depression because this study did not target depression. We did not include the subscales of sympathetic arousal and upper respiratory symptoms due to not finding change over time in these subscales in previous studies with this population. Validation studies have shown convergent validity with specific subscales and overall divergent validity with anxiety (Carlson & Thomas, 2007). Response options for the 41 items we included were on a scale from 0–4; higher mean scores indicate worse symptoms.

The Perceived Stress Reactivity Scale (PSRS) is a 23-item measure of stress-reactivity from work overload, social conflicts, failure, social evaluation, and prolonged reactivity (i.e., difficulty relaxing after a high workload day) (Schlotz et al., 2011). Based on cognitive interviews that we conducted with 33 clergy, we changed the PSRS response options from having only three options to four to promote response variability. We also edited some response options and some item stem wording (e.g., we changed the word “argue” to “disagree,” because clergy indicated “argue” conveys anger and that they would not endorse it). Further, we created two additional items of our own, for a total of 25 items. These new items included changing “When I have conflicts with others” to two items: “conflicts with congregants or colleagues” and “conflicts with friends or family members.” We also changed “When I do not attain a goal” to two items: “a work-related goal” and “a personal goal.” In addition, we allowed participants to indicate “not applicable” to each item, because during the cognitive interviews, many clergy said that the situation in various items did not pertain to them. Response options were on a scale from 0–3, and we scored items such that higher scores indicate more difficulty in reacting to stress.

To measure anxiety, we used the anxiety portion of the Hospital Anxiety and Depression Scale-Anxiety-HADS (Zigmond & Snaith, 1983). An example item is: “Over the past two weeks, how often have you been bothered by any of the following problems? I feel restless as if I have to be on the move.” The seven items have response options on a scale of 0–4, for a scale range of 0–28. We considered scores of 8 and above to indicate probable anxiety (Bjelland et al., 2002).

For the purposes of describing the sample, the survey included demographic items for gender, age, race, Hispanic/Latino ethnicity, and education. We included financial stress (“How stressful is your current financial situation for you? Not at all, slightly, moderately, very, and extremely stressful”), marital status, having children of any age living at home or not (yes/no), and an indicator of rural/urban work location. In the adjusted models, we control for two variables which may relate to stress: gender and years of experience in ministry (“How many years have you been in ministry full or part-time for which you were paid a salary?”). In addition, we included the Clergy Occupational Distress Index to describe stressors at Time 1, using a time frame of the past 4 weeks. This measure has 5 items, including “how often have you experienced stress because of the challenges you have in this organization/congregation” and “how often have the people in your congregation made too many demands on you” (Frenk et al., 2013).

#### Heart Rate Variability Measurement

Heart rate was measured using continuous electrocardiographic (ECG) recording sampled at a rate of 1,000 Hz. Participants were fitted with an eMotion Faros 180 ambulatory heart rate recording device (Bittium) connected by electrode leads to two pre-gelled (Ag/AgCl) disposable Ambu BlueSensor wet-gel ECG electrodes attached beneath the right clavicle and left ribcage. The ambulatory recording device was worn for 24 h immediately following the intervention workshop and at 12-weeks post-workshop (or, for control participants, at 0 weeks and 6–8 weeks), during which time participants proceeded with their usual daily routines and sleep activities.

Using the Mindware HRV Analysis (Version 3.0.3) software, the 24-h ECG data were partitioned into 300-s segments that were linearly detrended, and subject to a Hamming window. Each segment was scanned for artifacts according to accepted standards (Berntson et al., 1990). Segments with artifacts in excess of 10% were excluded. Interbeat intervals (IBIs) were calculated as the time between successive R-peaks. The root mean square of successive differences (RMSSD) was used as a time domain-based index corresponding to parasympathetic regulation of the heart. The RMSSD is less affected by breathing and is therefore a suitable outcome measure in ambulatory studies (Penttila et al., 2001).

Following recommendations for the detection of circadian rhythmicity (Refinetti et al., 2007), 5-min segments across 24 h of recording were subject to a cosinor analysis using the Cosinor package for R (R Development Core Team, 2011). Two individual-level cosine function parameters were estimated by linear models with ordinary least square estimations to quantify the circadian variability parameters: 1) midline estimating statistic of rhythm (MESOR) defined as the rhythm adjusted 24-h mean, and 2) amplitude, defined as the distance between MESOR and the maximum of the cosine curve (i.e., half the extent of rhythmic change in a cycle). We assumed periods of 24 h.

#### In-depth Interviews

In-depth interviews with questions on acceptability and feasibility were conducted with a subset of participants. We selected some participants with high uptake and some with low uptake of the practices based on text message data. Interviews were conducted over the phone for 30–60 min using a semi-structured design. One team member conducted content analysis (Hsieh & Shannon, 2005). Interviewees were not additionally compensated for this interview.

All procedures were approved by the Duke University Campus Institutional Review Board and all participants gave informed consent.

### Statistical Analysis

The primary aim of this study was to assess feasibility of the intervention and to collect preliminary outcome data to inform sample size calculations for a larger, fully powered study of intervention effectiveness. This study was not designed or powered to determine preliminary efficacy or effectiveness, thus all quantitative analysis is descriptive and no formal hypothesis testing was conducted (Lancaster et al., 2004). The focus of the quantitative analysis was on outcome summaries and their precision via means, standard deviations, and confidence intervals.

We reported summary statistics of participants’ demographic and occupational characteristics at Time 1, namely means and standard deviations for the continuous variables (age and experience in ministry); counts and percentages for the remaining variables). We examined baseline differences of the outcome variables with an ANOVA test across the 4 arms. For all outcome variables, we reported the means and standard deviations of these continuous variables at Time 1 and Time 3; we compared the means within each intervention arm between Time 1 and Time 3 by Student’s t test.

Standardized mean differences (i.e., Cohen’s *d*) and associated 95% and 75% CIs were calculated to explore the range of effect sizes likely to be observed on surveys and HRV within an adequately powered trial. Effect sizes were calculated as the mean difference from Time 1 to Time 3 and also from Time 1 to Time 2 with the pooled standard deviation being the denominator. Effect sizes were calculated within each treatment arm over time. Survey statistical analyses were conducted using Stata (Version 16.1). Standard mean differences and confidence intervals were visualized using Microsoft Excel.

## Results

### Participation and Preferences

A total of 71 participants enrolled with the intent to participate in an intervention of their choosing based on the described intervention content, dates, and location. The intervention arm that was preferred by most participants was Stress Proofing (n = 29, 41%), followed by the Examen (n = 17, 24%), MBSR (n = 13, 18%), and Centering Prayer (n = 12, 17%).

Demographic characteristics are reported in Table 1. The mean score on a measure of clergy stressors, the Clergy Occupational Distress Index, was 6.6. To compare, other studies have reported means of 11.0 for a nationally representative sample of clergy (n = 879) and 11.8 for clergy of Protestant denominations (n = 843) (Frenk et al., 2013).

**Table 1:** Participant characteristics by intervention arm

	Combined (n = 71) n (%) or M (SD)	MBSR (n = 13) n (%) or M (SD)	Stress Proofing (n = 29) n (%) or M (SD)	Daily Examen (n = 17) n (%) or M (SD)	Centering Prayer (n = 12) n (%) or M (SD)
Gender					
Male	41 (57.7)	7 (58.3)	12 (41.4)	13 (76.5)	8 (66.7)
Female	30 (42.3)	5 (41.7)	17 (58.6)	4 (23.5)	4 (33.3)
Age	52.2 (SD 10.3)	52.3 (SD 10.0)	53.1 (SD 9.3)	50.5 (SD 12.1)	52.2 (SD 11.2)
Experience in vocational ministry (in years)	16.2 (SD 9.6)	18.6 (SD 8.0)	16.5 (SD 10.2)	16.4 (SD 9.1)	12.8 (SD 10.6)
Race					
White, single racial	63 (92.6)	11 (91.7)	27 (93.1)	17 (100)	8 (72.7)
Black/African American, single racial	2 (2.9)	1 (8.3)	0 (0)	0 (0)	1 (9.1)
Asian American/Pacific Islander, single racial	2 (2.9)	0 (0)	0 (0)	0 (0)	2 (18.2)
Bi-/multi-racial	1 (1.5)	0 (0)	1 (6.9)	0 (0)	0 (0)
Education					
Bachelor’s degree or less	10 (14.3)	1 (8.3)	4 (13.8)	2 (11.8)	3 (25.0)
Master’s degree	55 (78.6)	11 (91.7)	25 (86.2)	11 (64.7)	8 (66.7)
Doctoral degree	5 (7.1)	0 (0)	0 (0)	4 (23.5)	1 (8.3)
Financial stress					
Extremely stressful	2 (2.9)	0 (0)	2 (6.9)	0 (0)	0 (0)
Very stressful	10 (14.3)	2 (16.7)	2 (6.9)	4 (23.5)	2 (16.7)
Moderately stressful	23 (32.9)	5 (41.7)	10 (34.5)	4 (23.5)	4 (33.3)
Slightly stressful	28 (40.0)	5 (41.7)	11 (37.9)	8 (47.1)	4 (33.3)
Not at all stressful	7 (10.0)	0 (0)	4 (13.8)	1 (5.9)	2 (16.7)
Marital status					
Never married or widowed	6 (8.6)	1 (8.3)	1 (3.5)	2 (11.8)	2 (16.7)
Married	56 (80.0)	10 (83.3)	23 (79.3)	14 (82.4)	9 (75.0)
Divorced or separated	8 (11.4)	1 (8.3)	5 (17.2)	1 (5.9)	1 (8.3)
Children at home (Yes)	33 (47.1)	8 (66.7)	11 (37.9)	7 (41.2)	7 (58.3)
Rural/urban status					
Rural or open country	18 (26.1)	4 (33.3)	5 (17.9)	5 (29.4)	4 (33.3)
Town or village of less than 10,000 people	21 (30.4)	3 (25.0)	8 (28.6)	4 (23.5)	6 (50.0)
In or around city of 10,000–249,000 people	22 (31.9)	3 (25.0)	10 (35.7)	7 (41.2)	2 (16.7)
In or around city of 250,000 or more people	8 (11.6)	2 (16.7)	5 (17.9)	1 (5.9)	0 (0)
Clergy Occupational Distress Index (CODI)	6.6 (SD 3.2)	8.5 (SD 3.6)	6.2 (SD 2.9)	7.2 (SD 3.5)	5.1 (SD 2.1)

### Intervention Uptake

Mean daily response rates to the text messages ranged from 73 to 92% for 12 weeks (see Table 2). Participation in all four interventions was high. The Daily Examen was the most frequently practiced with 60% of participants practicing on 80% of days, and when practiced, it was for an average of 11.3 min, which falls within the recommended time range. MBSR was the least practiced per day with 43% conducting formal MBSR practice on 50% of days, for an average of 22.6 min when practiced, although the recommended range was 45–60 min. On average, participants practiced within the recommended number of minutes for Stress Proofing and Centering Prayer. No harmful effects were reported.

**Table 2:** Frequency and minutes of stress reduction practice by intervention

	MBSR	Daily Examen	Stress proofing	Centering prayer
Number of participants included in text data*	7	10	14	4
Number of days with text data**	588	840	1,176	336
Average percentage of participants providing text data per day	72.6%	92.1%	79.3%	81.3%
Average percentage of participants reporting doing any practice per day	47.3%	68.9%	60.4%	56.5%
Percent of participants practicing at least 80% of the days (reported practice)	14.3%	60%	14.3%	0%
Percent of participants practicing at least 50% of the days (reported practice)	42.9%	80%	71.4%	75%
Percent of participants practicing less than 33% of the days (reported practice)	28.6%	20%	14.3%	0%
Average number of minutes practiced per day (among those who reported any practice)	24.5 min	11.3 min	15.8 min	20.3 min
Number of recommended minutes of daily practice	45–60 min	10–15 min	10–25 min	20 min

Data were available for the second cohort of each intervention only. Data were collected for 84 days for MBSR, Stress Proofing, and the Daily Examen, and 83 days for Centering Prayer

### In-depth Interview Results

We conducted in-depth interviews with 5–7 participants per arm. Themes are depicted in Table 3. Overall, all four interventions had acceptable learning content and practices. They were also all feasible in terms of the workshop attendance and practice uptake. 100% of participants in each arm reported that they would recommend the intervention to fellow clergy, despite how busy clergy are. Barriers to feasibility included being too busy to find time to practice when stress is highest, and, for the Daily Examen, finding it hard to practice when mentally fatigued. Barriers to acceptability were not having a spiritual emphasis in Stress Proofing and finding the quiet practices of Centering Prayer and the Daily Examen distracting to perform during a group workshop.

**Table 3:** Feasibility and acceptability themes that emerged from the in-depth interviews by intervention arm

	Feasible – positive	Feasible – negative	Acceptable – positive	Acceptable — negative
MBSR (*n* = 6)	Weekly online sessions worked well Can work informal MBSR practice into even unpredictable and busy days (e.g. while walking) Some of the skills (e.g. awareness of breath) felt easily achievable	30–45 min/day of formal practice was too much for some	Instructors were good at balancing the class between practice and discussion Class was often relaxing Positive mentions of specific skills learned, including body scan, breath awareness, and loving kindness meditation MBSR made some more in tune with their body and how it is used to serve God	Were taught many skills and some participants noted which ones (e.g. walking meditation) they did not adopt
Stress Proofing (*n* = 7)	Practice was readily incorporated into daily life Could practice throughout the day when needed	Not enough time to do the practice when stress is high	Instructor was engaging Information on stress response was helpful Nice balance of education and practice Breathing exercises were good Trying minor stressors was good	Not enough of a spiritual emphasis Uncomfortable touching other participants during exercises
Daily Examen (*n* = 5)	15 min was practical to incorporate into a daily routine The book and phone app were helpful	If mentally or physically fatigued, could be hard to do this practice	Instructors were interesting Liked practicing with a community (the workshop participants) Liked that the instructors regularly do the practice Fit with wanting structure to one’s prayer life Was able to share with congregants	Hard to practice in a group Workshop included information that wasn’t needed Not enough visuals and notes
Centering Prayer (*n* = 5)	Location and date of the workshop worked well 20 min on some days felt possible	20 min every day did not feel possible	Instructor (who was a pastor) was interesting, helpful, relatable, and compassionate The instructor bore “powerful witness” to the power of the practice Appreciations for contemplative spiritual practices like this one Some felt “closer to the Lord” and experienced a “more prayerful attitude”	Distracting to sit quietly for so long in a group

### Survey and HRV Results

The survey response rates for participants were 100% at Time 1, 93% at Time 2, and 92% at Time 3. Not all participants were asked to contribute HRV data; we used complete pairs of HRV data from 38 participants. Table 4 reports the means and standard deviations of the survey and HRV outcomes.

**Table 4:** Within-group comparisons of unadjusted means and standard deviations for each outcome over time

	Time 1		Time 3	
	Sample size	Mean (SD)	Sample size	Mean (SD)
C-SOSI stress symptoms, scale range 0–4	70		58	
MBSR	12	1.02 (0.46)	11	0.70 (0.58)
Stress Proofing	29	0.90 (0.50)	29	0.55 (0.36)
Daily Examen	17	0.65 (0.42)	16	0.51 (0.38)
Centering prayer	12	0.40 (0.27)	9	0.39 (0.27)
Stress reactivity, scale range 0–75	67		55	
MBSR	12	41.2 (10.6)	10	36.2 (9.3)
Stress Proofing	29	40.2 (12.2)	27	34.8 (9.4)
Daily Examen	15	38.7 (11.2)	16	34.1 (7.4)
Centering Prayer	11	32.2 (7.2)	9	26.9 (8.4)
HADS anxiety symptoms, scale range 0–21	70		58	
MBSR	12	6.25 (3.17)	11	4.21 (3.20)
Stress Proofing	29	4.72 (3.30)	29	3.36 (2.24)
Daily Examen	17	4.65 (3.06)	16	2.94 (1.81)
Centering prayer	12	2.92 (2.11)	9	3.44 (1.88)
MESOR RMSSD	38		38	
MBSR	6	34.8 (16.7)	6	42.0 (22.2)
Stress Proofing	14	32.0 (21.6)	14	26.6 (16.4)
Daily Examen	11	40.7 (24.1)	11	42.9 (16.8)
Centering prayer	7	35.9 (28.6)	7	42.7 (34.6)
Amplitude RMSSD	38		38	
MBSR	6	14.7 (13.5)	6	17.8 (12.1)
Stress Proofing	14	13.4 (15.4)	14	8.2 (7.5)
Daily Examen	11	15.5 (15.4)	11	15.9 (11.8)
Centering prayer	7	14.9 (20.9)	7	22.4 (25.5)

As shown in Table 4, mean unadjusted survey scores changed in the expected direction from Time 1 to Time 3 for MBSR, Stress Proofing, and Daily Examen participants; results were mixed for Centering Prayer participants. Mean unadjusted HRV scores changed in the expected direction from Time 1 to Time 3 for MBSR and Centering Prayer participants, showed little change for Examen participants, and changed in the unexpected direction for Stress Proofing participants.

**Fig. 1:**
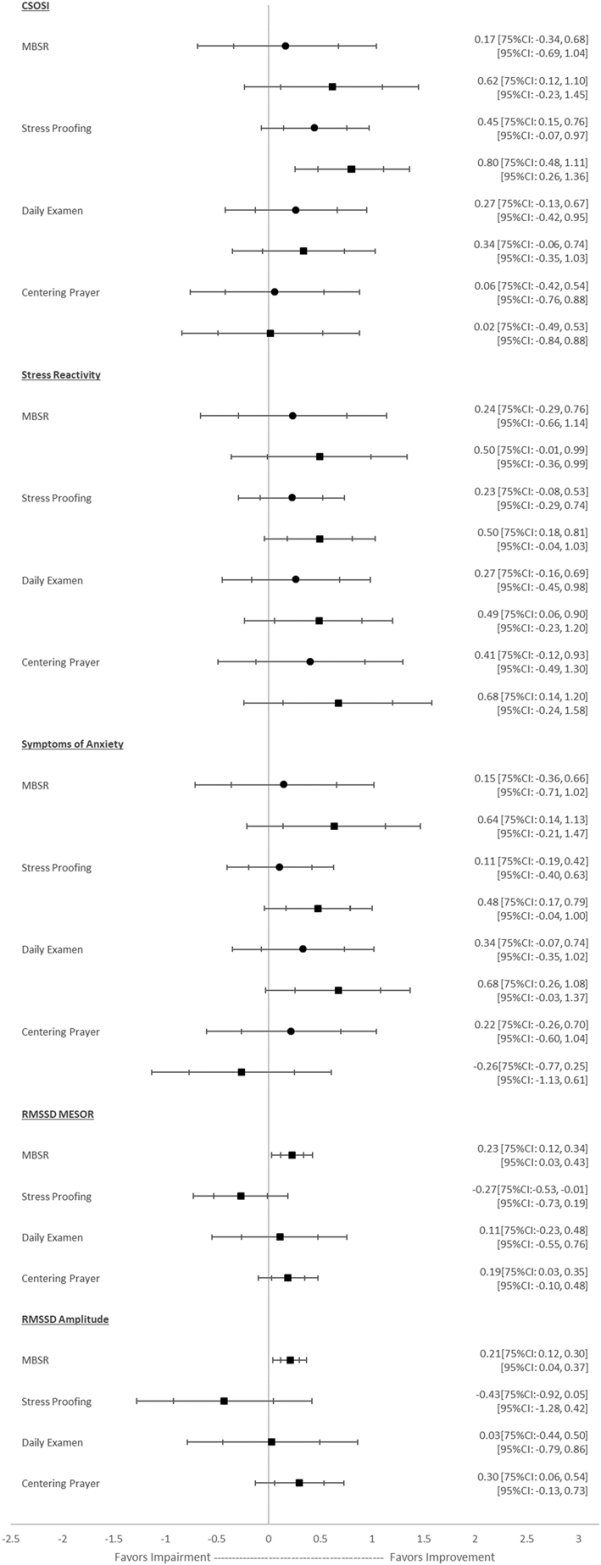
Within-group estimates and confidence intervals for five outcomes by treatment arm. The circles indicate standardized mean differences at 3 weeks compared to baseline and the boxes indicate standardized mean differences at 12 weeks compared to baseline, with the bars showing 75% and 95% CIs. Standardized mean difference values of 0.2, 0.5., and 0.8 refer to small, medium and large effects (Cohen, 1992)

Figure 1 depicts standardized mean differences (which are effect sizes) with 75% and 95% confidence intervals when comparing within-group change from Time 1 to Time 2 and from Time 1 to Time 3. Participants in the MBSR, Stress Proofing, and Examen arms tended to report improvements in symptoms of stress, stress reactivity, and anxiety at 12 weeks with medium-to-large effect sizes with 75% CIs that generally exceeded 0. The effect of Centering Prayer on survey-based measures of stress and anxiety were variable. The effect of interventions on HRV MESOR and amplitude also varied. MBSR and Centering Prayer demonstrated improvements over time, the Examen coefficients were close to 0 and with 75% CIs that did not exceed 0, and Stress Proofing coefficients were in the unexpected direction.

## Discussion

We performed a Phase-II feasibility pilot study to determine which of four potential stress reduction interventions, which included two spiritual practices, were acceptable, feasible, and potentially efficacious among clergy to proceed to a Phase-III efficacy trial. Each intervention was feasible and acceptable to United Methodist clergy. Engagement was high across interventions with a majority of participants reporting engaging in intervention practice multiple days per week. Moreover, the range of effect sizes comparing within-intervention pre-test to 12-week post-test scores on symptoms of stress and stress reactivity encompassed thresholds for practical significance (mean difference > 0.41; Ferguson, 2009). With the exception of Centering Prayer, the point estimate of effect for symptoms of stress and stress reactivity were at or above this threshold of practical significance which supports further examination in a Phase-III efficacy trial. The effect of interventions on HRV MESOR and amplitude were mixed, with MBSR and Centering Prayer resulting in an appreciable change relative to control.

The primary reason to offer four different programming options to clergy was to discover which interventions they would and would not find acceptable and feasible, and to identify barriers that might inform intervention redesign before proceeding to an adequately powered and resource-intensive trial. We believed participating in the intervention of one’s preference may result in higher engagement, which may lead to better outcomes. We thus allowed clergy to enroll in the intervention of their choosing, and we considered enrollment size to be an indicator of acceptability. Stress Proofing was the most popular, followed by the Daily Examen. Clergy may have been particularly attracted to Stress Proofing because it offered a two-night stay in a retreat center and few clergy had experience with its stress reduction practices. In contrast, all other interventions did not offer an overnight stay. Centering Prayer had the lowest enrollment, which may have been due to UMC clergy in North Carolina already having had opportunities to learn Centering Prayer through a non-profit organization and due to the slightly less desirable and accessible locations offered.

Interview comments on acceptability across the interventions were strongly positive. Participants noted that the two spiritual practices matched their desired prayer life and helped them feel closer to God. Interestingly, one participant also commented that MBSR connected them to their body and that this had a desirable outcome of helping them consider how their body is used to serve God. No one mentioned this connection for Stress Proofing, which also sought to put people in touch with their bodies. Instead, one participant who completed Stress Proofing expressed a wish for spiritual content.

We allowed clergy of all physical health states to participate. United Methodist clergy have previously been documented having above-average rates of chronic diseases, including hypertension, diabetes, arthritis, angina, and asthma (Proeschold-Bell & LeGrand, 2010), although they do not always perceive the physical toll on their health (Proeschold-Bell & LeGrand, 2012). An important question is whether participants at risk of chronic disease find a stress management intervention to be feasible. In the current study, all interventions appeared feasible for clergy. Regular practice of the Daily Examen appeared especially feasible, with 80.0% practicing it on at least half of the days across 12 weeks. MBSR had the lowest daily practice of the four interventions, with 42.9% practicing it at least half of the days, which is nevertheless a remarkable behavior change for many participants.

MBSR practice is likely to yield good outcomes; of the four interventions tested, it has the most robust evidence base with prior outcome studies reporting reduced symptoms of anxiety (Smith et al., 2015; Zhang et al., 2015), reduced symptoms of depression (Goldin & Gross, 2010), decreased stress (Burton, 2017), and improvement in sleep quality (Karaca & Sisman, 2019), and being effective among participants with high anxiety and poor sleep quality at baseline (Brown et al., 2020). We do not yet have a good understanding about the dose (i.e., frequency and number of minutes engaged in practice) needed to experience improvement in stress. In the current study, the number of minutes practiced (22.6) was lower than what MBSR outcome studies have tested. In one meta-analysis, the range of minutes practiced was 60–120 min (Veehof et al., 2016). However, fewer minutes of daily practice may be beneficial. For example, Smith et al. (2015) found decreased perceived stress, decreased anxiety, increased awareness, and increased acceptance with 15–25 min of daily practice.

A study objective was to identify modifications that could be made to improve the interventions before proceeding to an adequately powered trial. We recommend offering an overnight stay to allow for travel time and enough calming space to practice stress management skills. For Stress Proofing, we recommend that clergy learn the skills without engaging in physical contact with one another, and incorporating reasonable spiritual concepts into activities, such as the sacredness breath. In addition, the Stress Proofing content was broad and heavily didactic; we recommend starting the workshop with an activity, being clearer on which activities to regularly practice, and cutting back on the amount of time spent teaching the physiology of stress. For Centering Prayer, some participants found it hard to sit for 20 min without distraction in a group setting, while others enjoyed practicing in a group; perhaps expectations could be set in advance. For the Examen, reports of the two post-workshop sessions using a web platform at 2 and 4 weeks later were highly positive; we recommend considering this structure across interventions.

We collected data on practice adherence using text messages. Although we were initially concerned that a daily text message would be perceived as annoying, participants nearly universally indicated that they welcomed the daily message as a reminder and accountability structure, such that even programs not interested in evaluation should consider including text messages. We recommend sending the message at noon and again at 4 pm for non-responders. We recommend personalizing the text messages with the participant’s name, and varying an intro message (e.g. “Peace be with you!” every few weeks.)

We collected outcome data on a small sample of participants in this pilot study to determine the feasibility of trial procedures. We found that the 3-week survey assessment did not contribute much unique information and therefore we do not plan to collect 3-week data in the trial. We found that text messages were best sent daily, as opposed to every two days.

We also used the outcome data to assess the likelihood of change in symptoms for each intervention, with particular interest in the spiritual practices because relatively few studies of spiritual practice interventions for stress reduction exist. For the Examen, we found promising changes in stress and anxiety symptoms and stress reactivity, but neutral changes for HRV. For Centering Prayer, we found promising change patterns for HRV and stress reactivity, but neutral to increased stress and anxiety symptoms. In contrast, another study of Centering Prayer found decreased anxiety symptoms using a different measure for participants who practiced 20 min six times a week (Hayter et al., 2019).

Stress Proofing showed statistically significant improvements in stress symptoms, which is consistent with other stress inoculation training intervention studies, for example among pregnant women who report reductions in perceived stress (Khorsandi et al., 2016). However, Stress Proofing showed deteriorations in stress response based on the HRV measures. For future tests of Stress Proofing, we recommend increased focus on and motivation for the breathing and physical practices that can be incorporated multiple times per day. For MBSR, change patterns were consistently positive. As a point of reference, the decrease in anxiety symptoms was small (-2.0 points), but akin to other studies using the same anxiety measure as an outcome for MBSR interventions (3.4 points (Smith et al., 2015); 1.9 points (Dvorakova et al., 2017)).

We evaluated the likelihood that interventions would produce change in symptoms of stress and stress reactivity. Interventions were considered promising and moved to full trial if the point estimate of effect was close to recommendations for minimum practically significant effects (i.e. mean difference ≥ 0.41; Ferguson, 2009). Adopting these criteria, MBSR, Stress Proofing, and the Examen, but not Centering Prayer, were considered interventions with particular promise to improve stress management of clergy and proceeded to Phase-III efficacy testing in the ongoing Selah trial.

The stress management interventions we evaluated produced less reliable change in long-term HRV parameters. RMSSD is an indirect indicator of the strength of the parasympathetic nervous system on heart rate and correlates well with high frequency HRV (Malik, 1996). We chose to include RMSSD given that it may serve as a proximal indicator for integration of brain mechanisms that guide flexible control over behavior with peripheral physiology and may provide an important window into understanding stress and health (Thayer et al., 2012). Moreover, long-term measures of RMSSD have been associated with markers of stress at work among adults between 35 and 44 years of age (Loerbroks et al., 2010). It has been recognized that perceived and objective measures of stress assess different aspects of the psychobiological sequelae that is stress, with multi-method assessments being favored (Weckesser et al., 2019). In the trial, we will use multi-method assessments including RMSSD, but the sample size was not large enough in this pilot study to use RMSSD measures to inform which interventions to proceed to trial.

## Limitations

This study has several limitations, most notably the small sample size of the intervention groups and the lack of a robust control group. Because we only used 5 of the 8 C-SOSI subscales, we cannot assume the same degree of stress symptom validity as in the C-SOSI development studies. We recruited a convenience sample of clergy; our sample may lack the full diversity of clergy experiences. Findings are most generalizable to United Methodist clergy but may be relevant to clergy of other Christian denominations. We did not randomly assign participants to intervention arms because we believe that participant preference is important to sustain daily stress reduction practices. This introduces selection bias into intervention arms and conflates intervention effects with expectancy effects. As such, results must be interpreted within intervention arm rather than between arms. Study strengths include collecting a variety of kinds of acceptability and feasibility data, collecting survey and physiological data, and collecting daily participation data for 12 weeks.

## Conclusion

In conclusion, the current study reports on Phase II preliminary testing of four potential stress reduction interventions, including two under-studied spiritual practices that may align with the values and thus preferences of Christian clergy and individuals. All four interventions were acceptable and feasible for clergy, and have the potential of appealing to busy professionals who are called to their work more broadly. The preliminary testing reported here is critical to inform trial conduct, enhance intervention redesign, and improve resource expenditure by ensuring that only well-informed interventions proceed to Phase III efficacy trials, including the Selah Phase III trial.

## Data Availability

The datasets generated during the current study are not publicly available, but de-identified data will be made available on reasonable request where such requests are compliant with receipt of ethical approval from the sending and receiving hosts’ institutional ethics review boards.
